# Influencing factors of humanistic care ability and its dimensions among mental health workers during the COVID-19 pandemic: an online cross-sectional study

**DOI:** 10.1186/s12888-023-04656-5

**Published:** 2023-03-21

**Authors:** Xiaolin Liu, Hongjin Zhu

**Affiliations:** 1 Rehabilitation Department, Jinzi Mountain Hospital of Chongqing Mental Health Center, No.102, Jinzi Mountain, Jiangbei District, Chongqing, 401147 China; 2 Nursing College of Chongqing Medical University, Chongqing, China

**Keywords:** Cognition, Courage, COVID-19, Psychological capital, Personality, Healthcare workers

## Abstract

**Background:**

In psychiatric services, humanistic care ability significantly affects the quality of the therapeutic relationship and thus affects the therapeutic outcomes for patients. Mental health workers may be confronted with more obstacles in humanistic care during the COVID-19 pandemic wherethe authors aimed to explore the capacity level of humanistic care among mental health workers and its potential influencing factors.

**Method(s):**

An online cross-sectional survey was conducted among 262 mental health workers working in Chongqing, China, from December 2020 to January 2021. Data were collected by the Caring Ability Inventory (CAI), the Psychological Capital Questionnaire (PCQ-24), the Eysenck Personality Questionnaire-Revised, and the Short Scale for Chinese (EPQ-RSC). Multiple linear regression analysis was used to explore the influencing factors of humanistic care ability.

**Results:**

Mental health workers’ humanistic care ability is at a low level, with a score of 186.47 ± 21.34. Psychological capital is positively associated with humanistic care ability (*β*[95%*CI*] = 0.41 [0.46–0.77], *p* < 0.001), and its two dimensions (cognition: *β*[95%*CI*] = 0.51 [0.30–0.47], *p* < 0.001; patience: *β*[95%*CI*] = 0.48 [0.17–0.28], *p* < 0.001). Psychoticism is negatively associated with humanistic care ability (*β*[95%*CI*] = -0.28 [-5.18 - -2.51], *p* < 0.001) and its three dimensions (cognition: *β*[95%*CI*] = -0.12 [-1.57 - -0.17], *p* < 0.05; courage: *β*[95%*CI*] = -0.17 [-1.7 - -0.32], *p* < 0.01; patience: *β*[95%*CI*] = -0.19 [-1.33 - -0.36], *p* < 0.01). Extroversion is positively associated with humanistic care ability (*β*[95%*CI*] = 0.19 [0.69–2.08], *p* < 0.001), and its two dimensions (cognition: *β*[95%*CI*] = 0.19 [0.32–1.05], *p* < 0.001; courage: *β*[95%*CI*] = 0.27 [0.5–1.23], *p* < 0.001). Neuroticism is negatively associated with humanistic care ability (*β*[95%*CI*] = -0.13[-1.37 - -0.19], *p* < 0.01) and its one dimension (courage: *β*[95%*CI*] = -0.25 [-0.98 - -0.35], *p* < 0.001).

**Conclusion(s):**

The research has found that the humanistic care ability of mental health workers is at a low level, and the psychological capital and personality traits are significant factors influencing the humanistic care ability and its sub-dimensions. Interventions to improve the psychological capital of mental health workers or to promote the change of personality traits they want are recommended, thereby to promote humanistic practice.

## Background

The humanistic care is the ability to listen to the needs and desires of patients, to understand patients’ emotions, to communicate with patients, and to feel the value of life to develop therapeutic relationships [1]. It emphasizes the caring for the ‘whole person’, which is, being aware of the physical, cognitive, psychological, emotional, social, and spiritual dimensions of a person, as compared with the narrow focus on the physical elements of disease that often dominates medical practice [2]. The humanistic care ability significantly affects the professional performance of medical staff, the quality of patients’ life [3, 4], and the costs and outcomes of health care [5, 6]. Therefore, the entire health system emphasizes the need to promote humanistic caring, including in the clinical field, education, management, and public policies [7]. For example, in 2010, the Ministry of Health of China launched the ‘High-Quality Nursing Service’ project in the national health system, requiring clearly that the “patient-centered” service concept and humanistic care should be integrated into the clinical work [8]. In 2016, the “Healthy China 2030” planning outline once again clarified that “The industry needs to strengthen the humanistic care of medical services and to build a harmonious doctor-patient relationship [9]. It can be seen that the humanistic care ability of medical staff is an indispensable component of their professional practices. Medical staff need to integrate the value of the individual, care, warmth, and compassion into the daily care work [10, 11] to better develop humanistic care ability and maintain humanistic practices.

Although the importance of humanistic care is emphasized both at the theory and policy level, and education and training programs have been proposed in previous studies [12, 13], medical staff has not been able to translate these results into daily clinical work, and their humanistic care ability isis still at a relatively low level [14]. One of the reasons for this result is various clinical challenges, such as time constraints, work-related or personal stress, organizational culture, and burnout factors [15]. Notably, these challenges may be more significant during the COVID-19 pandemic, especially in the field of mental healthcare. The direct impact of the virus and measures such as lockdown restrictions have caused huge psychological problems among different sub-populations, including confirmed or suspected COVID-19 patients [16], mental patients [17], the general public, and medical staff [18]. Deng et al. [18] also noted that the mental health of the general population tends to deteriorateafter the peak of the epidemic. This increases the demand for mental health services. Mental health workers are facing more workload and pressure. Patients and their relatives may receive less attention or humanistic care from these institutions and their staff, which is not conducive to their full recovery. Furthermore, some specific stressors in mental healthcare settings, such as stigma [19], higher frequency of violence [20], under-funding for mental health services [21], and insufficiency of professional institutions and practitioners [22] have also hindered the humanistic care practices of mental health workers.

In psychiatric services, the quality of the therapeutic relationship has a significant impact upon the therapeutic outcomes for the patients [23]. It is increasingly important to focus on and cultivate the humanistic care ability of mental health workers, and to integrate the “patient-centered” service concept and humanistic care into clinical settingsHowever, during the pandemic, researchers have mostly focused on the mental health of healthcare workers and seem to pay less attention to humanistic care ability. The purpose of this study is to explore the humanistic care ability of mental health workers during the COVID-19 pandemic, to clarify its broader influencing factors, and to provide a new direction for improving thehumanistic care in mental health services. For these purposes, the authors propose the following hypotheses: Hypothesis 1: During the COVID-19 period, the humanistic care ability of mental health workers is low; Hypothesis 2: The social-demographic factors affect the humanistic care ability of mental health workers; and Hypothesis 3: Personality traits and psychological capital are predictors of humanistic care ability.

## Methods

### Design, participants, and procedures

This study has an online cross-sectional design. A non-random sample of mental health workers were selected from December 2020 to January 2021 in Chongqing, China. The selection includes mental health workers, such as doctors, nurses, or medical technicians, who were willing to participate and received a professional certificate. The research excludes mental health workers who were unwilling to participate and temporarily absent due to illness, maternity leave, and personal leave.

The G*Power 3.1.9.2 program was used to estimate the sample size. A sample size of 164 was required to obtain a medium effect size (f2 = 0.15) for multiple linear regression analysis, at a two-sided significance threshold of 0.01 and a power (1 − *β*) of 0.99. The sample size required for the study was at least 181 based on the 10% dropout rate.

Questionnaires were distributed by managers to workers via the department’s We-Chat group, which is one of the most widely used social networking software in China. All items were mandatory to select to prevent missing data. To avoid duplication, each phone IP address could be allowed only once to visit and complete the survey. Surveys with suspected unreal answers (obvious logic contradictions, all answers the same to different questions) were excluded before data analysis.

### Measurements

Humanistic care ability was assessed with the Chinese version of the Caring Ability Inventory (CAI) [24], which includes three dimensions: cognition (14 items), courage (13 items), and patience (10 items). Each item was scored on a 7-point Likert scale ranging from 1 (strongly disagree) to 7 (strongly agree), and courage items were scored in reverse. The higher the total score, the higher the level of humanistic care ability. The Cronbach’s α in this study was 0.852.

Psychological capital (PsyCap) was assessed with the Chinese version of the Psychological Capital Questionnaire 24 (PCQ-24) [25], which consisted of four sub-scales: self-efficacy (6 items), hope (6 items), resilience (6 items), and optimism (6 items). Each item was scored on a 6-point Likert scale ranging from 1 (strongly disagree) to 6 (strongly agree). The higher the total score, the higher the level of psychological capital. The Cronbach’s α in this study was 0.933.

Personality traits were assessed with the Eysenck Personality Questionnaire-Revised, Short Scale for Chinese (EPQ-RSC) [26]. This scale includes 48 items and 4 dimensions: psychoticism (P), neuroticism (N), extroversion (E), and lie (L). This study analyses only the three dimensions of P, E, and N. The EPQ-RSC has well-established psychometric properties and is suitable for the measurement of the personality traits of Chinese mainland adults.

Participants’ social-demographic variables include hospital level, hospital nature, professional category, gender, age, marital status, education level, work years, work shift, work pressure, practice environment satisfaction, salary satisfaction, and work-family conflict.

### Data analysis

SPSS version 25.0 was used to analyse the data. Descriptive statistics are reported as frequency, percentage, mean, and standard deviations. Univariate analysis of humanistic care ability concerning categorical variables was examined by t-tests and one-way analyses of variance (ANOVAs). The univariate analysis of humanistic care ability with continuous variables was tested by Pearson’s correlation analysis. Independent variables with statistical significance in the univariate analysis were added into the multivariate analyses. The multiple linear regression analysis was used to identify the influencing factors of humanistic care ability. The statistical significance for all analyses was set to *p* < 0.05 (2-tailed).

## Results

### Social-demographic characteristics of the participants

Of the 298 returned questionnaires, 262 questionnaires were effective for analysis, and the effective return rate was 88%. The majority of the participants were female (75.6%), who weremarried (75.2%), and had an undergraduate degree or above (74.4%). The majority of mental health workers came from Grade A hospitals and specialist hospitals, at 73.3% and 89.3%, respectively. In terms of the professional category, nurses accounted for 64.5% of participants, which was noteworthy. Most participants were on work shifts (70.6%). 42.7% and 25.2% experienced high work pressure and work-family conflict, respectively. About one-third of the participants expressed dissatisfaction with the practice environment (26.3%) and the salary (31.3%). More details are shown in Table 1.

**Table 1:** Univariate analysis of humanistic care ability in relation to categorical variables

Variables	N (%)	Humanistic care ability (Mean [SD])			
		Overall	Cognition	Courage	Patience
**Gender**					
Male	64 (24.4)	182.58 (20.66)	73.59 (10.77)	52.45 (10.19)	56.5 (7.94)
Female	198 (75.6)	187.73 (21.45)	74.72 (10.73)	54.88 (9.78)	58.1 (7.12)
**Marital status**					
Unmarried	57 (21.8)	185.40 (21.98)	72.74 (10.67)	55.47 (9.04)	57.19 (7.32)
Married	197 (75.2)	186.77 (21.50)	74.92 (10.91)	54.02 (10.26)	57.83 (7.44)
Other	8 (3.1)	186.63 (12.33)	74.75 (4.40)	52.50 (6.82)	59.38 (5.29)
**Education level**					
Junior college or lower	67 (25.6)	188.31 (18.07)	75.19 (9.96)	54.43 (9.51)	58.69 (6.10)
Undergraduate degree or above	195 (74.4)	185.84 (22.36)	74.18 (11.00)	54.24 (10.08)	57.41 (7.71)
**Hospital level**					
Grade A	192 (73.3)	185.61 (20.83)	73.71 (10.55)	54.66 (9.51)	57.25 (7.20)
Grade B	49 (18.7)	192.22 (22.98)	77.59 (11.32)	54.82 (11.57)	59.82 (7.70)
Grade C	21 (8)	180.86 (20.27)	73.81 (10.13)	49.71 (8.64)	57.33 (7.26)
**Hospital nature**					
General Hospital	28 (10.7)	189.68 (22.79)	77.14 (10.62)	53.29 (11.08)	59.25 (6.74)
Specialty Hospital	234 (89.3)	186.09 (21.18)	74.12 (10.72)	54.41 (9.79)	57.56 (7.40)
**Professional category**					
Nurse	169 (64.5)	186.29 (21.04)	74.40 (10.54)	54.11 (10.10)	57.79 (7.26)
Doctor	80 (30.5)	186.33 (22.08)	74.33 (11.37)	54.46 (9.59)	57.54 (7.75)
Medical Technician	13 (5)	189.69 (21.96)	75.77 (9.77)	55.62 (10.16)	58.31 (6.20)
**Work shift**					
Yes	185 (70.6)	185.47 (22.48)	73.87 (11.41)	54.46 (9.99)	57.14 (7.89)
No	77 (29.4)	188.87 (18.21)	75.82 (8.82)	53.87 (9.80)	59.18 (5.61) ^*^
**Work years**					
≤ 5 years	65 (24.8)	188.00 (20.47)	74.34 (10.35)	55.38 (9.58)	58.28 (6.59)
6–10 years	75 (28.6)	183.89 (21.19)	73.61 (10.53)	53.39 (9.02)	56.89 (8.45)
11–15 years	58 (22.1)	183.50 (22.66)	73.60 (11.45)	52.88 (11.01)	57.02 (7.21)
≥ 16 years	64 (24.4)	190.63 (20.80)	76.28 (10.71)	55.52 (10.18)	58.83 (6.75)
**Work pressure**					
Low	11 (4.2)	193.36 (18.20)	80.36 (10.57)	52.27 (12.95)	60.73 (5.59)
Medium	139 (53.1)	188.53 (22.36)	75.01 (10.64)	55.83 (9.54)	57.69 (7.36)
High	112 (42.7)	183.23 (19.97)	73.16(10.69)	52.57 (9.83) ^*^	57.50 (7.47)
**Practice environment satisfaction**					
Dissatisfied	69 (26.3)	181.39 (22.29)	72.84 (11.66)	51.54 (10.49)	57.01 (7.54)
Neutral	124 (47.3)	185.85 (20.94)	73.49 (10.28)	55.10 (9.24)	57.26 (7.52)
Satisfied	69 (26.3)	192.67 (19.79) ^**^	77.75 (9.98) ^*^	55.59 (10.12) ^*^	59.32 (6.67)
**Salary satisfaction**					
Dissatisfied	82 (31.3)	182.28 (21.95)	72.20 (11.49)	53.34 (9.93)	56.74 (7.51)
Neutral	133 (50.8)	186.77 (19.54)	74.95 (9.61)	53.91 (9.50)	57.91 (6.87)
Satisfied	47 (17.9)	192.94 (23.78) ^*^	76.94 (11.82) ^*^	57.02 (10.77)	58.98 (8.22)
**Work-family conflict**					
Low	76 (29)	191.88 (21.33)	76.51 (10.64)	57.13 (9.41)	58.24 (6.98)
Medium	120 (45.8)	185.83 (21.04)	74.13 (10.31)	53.95 (10.01)	57.76 (7.39)
High	66 (25.2)	181.39 (20.77) ^*^	72.64 (11.34)	51.64 (9.61) ^**^	57.12 (7.72)

*P < 0.05, **P < 0.01, *** P < 0.001. SD, standard deviation

### T-tests or one-way ANOVAs of humanistic care ability about categorical variables

The practice environment satisfaction, salary satisfaction, and work-family conflict were significantly associated with humanistic care ability (*p* < 0.05). Practice environment satisfaction and salary satisfaction were significantly associated with cognition (*p* < 0.05). Work pressure, practice environment satisfaction, and work-family conflict were significantly associated with courage (*p* < 0.05). Work shift was significantly associated with patience (*p* < 0.05). The above results are shown in Table 1.

### Correlation analysis of humanistic care ability in relation to continuous variables

The average age was (35.16 ± 8.16) years. The mean scores of the humanistic care ability, cognition, courage, and patience was 186.47 (± 21.34), 74.44 (± 10.73), 54.29 (± 9.92), 57.74 (± 7.34), respectively.

The correlation analysis showed that psychological capital was significantly associated with humanistic care ability (overall: r = 0.573, *p* < 0.05; cognition: r = 0.595, *p* < 0.05; courage: r = 0.236, *p* < 0.05; patience: r = 0.477, *p* < 0.05). Psychoticism was significantly associated with humanistic care ability (overall: r = -0.411, *p* < 0.05; cognition: r = -0.328, *p* < 0.05; courage: r = -0.252, *p* < 0.05; patience: r = -0.376, *p* < 0.05). Extroversion was significantly associated with humanistic care ability (overall: r = 0.387, *p* < 0.05; cognition: r = 0.335, *p* < 0.05; courage: r = 0.305, *p* < 0.05; patience: r = 0.221, *p* < 0.05). Neuroticism was significantly associated with humanistic care ability (overall: r = -0.337, *p* < 0.05; cognition: r = -0.272, *p* < 0.05; courage: r = -0.323, *p* < 0.05; patience: r = -0.146, *p* < 0.05). The above results are shown in Table 2.

**Table 2:** Correlation analysis of caring ability in relation to continuous variables

	1	2	3	4	5	6	7	8	9
1 Humanistic Care ability	1								
2 Cognition	0.871^**^	1							
3 Courage	0.602^**^	0.182^**^	1						
4 Patience	0.820^**^	0.825^**^	0.131^*^	1					
5 Age	0.060	0.082	0.004	0.051	1				
6 PsyCap	0.573^**^	0.595^**^	0.236^**^	0.477^**^	0.053	1			
7 P	− 0.411^**^	− 0.328^**^	− 0.252^**^	− 0.376^**^	− 0.066	− 0.262^**^	1		
8 E	0.387^**^	0.335^**^	0.305^**^	0.221^**^	− 0.182^**^	0.294^**^	− 0.200^**^	1	
9 N	− 0.337^**^	− 0.272^**^	− 0.323^**^	− 0.146^*^	0.001	− 0.357^**^	0.103	− 0.163^**^	1
Mean	186.47	74.44	54.29	57.74	35.16	79.37	1.98	7.65	5.09
SD	21.34	10.73	9.92	7.34	8.16	13.96	1.52	2.86	3.51

PsyCap, psychological capital; P, psychoticism; E, extroversion; N, neuroticism; SD, standard deviation

*P < 0.05, **P < 0.01

### Multiple linear regression of the influencing factors of mental health workers’ humanistic care ability

In multiple linear regression analysis, humanistic care ability and its three dimensions were the dependent variables, and all possible predictors (p < 0.05 in univariate analysis) were added as independent variables. Table 3 shows the result of the multiple regression analysis. Psychological capital was positively associated with humanistic care ability (*β*[95%*CI*] = 0.41 [0.46–0.77], *p* < 0.001), and its two dimensions (cognition: *β*[95%*CI*] = 0.51 [0.30–0.47], *p* < 0.001; patience: *β*[95%*CI*] = 0.48 [0.17–0.28], *p* < 0.001). Psychoticism was negatively associated with humanistic care ability (*β*[95%*CI*] = -0.28 [-5.18 - -2.51], *p* < 0.001) and its three dimensions (cognition: *β*[95%*CI*] = -0.12 [-1.57 - -0.17], *p* < 0.05; courage: *β*[95%*CI*] = -0.17 [-1.7 - -0.32], *p* < 0.01; patience: *β*[95%*CI*] = -0.19 [-1.33 - -0.36], *p* < 0.01). Extroversion was positively associated with humanistic care ability (*β*[95%*CI*] = 0.19 [0.69–2.08], *p* < 0.001), and its two dimensions (cognition: *β*[95%*CI*] = 0.19 [0.32–1.05], *p* < 0.001; courage: *β*[95%*CI*] = 0.27 [0.5–1.23], *p* < 0.001). Neuroticism was negatively associated with humanistic care ability (*β*[95%*CI*] = -0.13 [-1.37 - -0.19], *p* < 0.01), and its one dimension (courage: *β*[95%*CI*] = -0.25 [-0.98 - -0.35], *p* < 0.001). The above results showed that psychological capital and personality traits were associated with humanistic care ability.

**Table 3:** Multiple linear regression analysis results, with humanistic care ability and three dimensions as the dependent variables

Model	Independent variables	B	SE	*β* (95%*CI)*	t	*P*
Humanistic care ability						
	(Constant)	139.898	8.426		16.603	0.000^***^
	Practice environment satisfaction	-1.374	1.555	− 0.05 (-4.44–1.69)	− 0.884	0.378
	Salary satisfaction	1.235	1.575	0.04 (-1.87–4.34)	0.784	0.434
	Work-family conflict	− 0.526	1.383	− 0.02 (-3.25–2.20)	− 0.380	0.704
	PsyCap	0.618	0.078	0.41 (0.46–0.77)	7.901	0.000^***^
	P	-3.846	0.677	− 0.28 (-5.18 - -2.51)	-5.684	0.000^***^
	E	1.383	0.354	0.19 (0.69–2.08)	3.909	0.000^***^
	N	− 0.780	0.298	− 0.13 (-1.37 - -0.19)	-2.615	0.009^**^
Cognition						
	(Constant)	41.063	4.062		10.109	0.000^***^
	Practice environment satisfaction	-0.694	0.789	-0.05 (-2.25–0.86)	-0.880	0.380
	Salary satisfaction	0.780	0.818	0.05 (-0.83–2.39)	0.954	0.341
	PsyCap	0.385	0.041	0.51 (0.30–0.47)	9.435	0.000^***^
	P	-0.866	0.356	-0.12 (-1.57 - -0.17)	-2.432	0.016^*^
	E	0.687	0.185	0.19 (0.32–1.05)	3.717	0.000^***^
	N	-0.104	0.152	-0.04 (-0.40–0.2)	-0.685	0.494
Courage						
	(Constant)	55.942	4.787		11.686	0.000^***^
	Work pressure	-0.733	0.935	-0.05 (-2.57–1.11)	-0.784	0.434
	Practice environment satisfaction	0.106	0.752	0.01 (-1.37–1.59)	0.141	0.888
	Work-family conflict	-1.042	0.740	-0.08 (-2.5–0.42)	-1.408	0.160
	PsyCap	0.015	0.042	0.02 (-0.07–0.1)	0.356	0.722
	P	-1.007	0.351	-0.17 (-1.7 - -0.32)	-2.869	0.004^**^
	E	0.863	0.186	0.27 (0.5–1.23)	4.632	0.000^***^
	N	-0.664	0.159	-0.25 (-0.98 - -0.35)	-4.184	0.000^***^
Patience						
	(Constant)	39.516	2.755		14.341	0.000^***^
	Work shift	0.508	0.765	0.04 (-0.1–2.02)	0.664	0.507
	PsyCap	0.227	0.028	0.48 (0.17–0.28)	7.995	0.000^***^
	P	-0.844	0.247	-0.19 (-1.33 - -0.36)	-3.418	0.001^**^
	E	0.151	0.129	0.07 (-0.1–0.41)	1.170	0.243
	N	0.095	0.107	0.05 (-0.12–0.31)	0.890	0.375

PsyCap, psychological capital; P, psychoticism; E, extroversion; N, neuroticism;

*P < 0.05, **P < 0.01, *** P < 0.001

Model performance of Humanistic Care ability: R2 = 0.480, adjusted R2 = 0.465, F = 33.208, p < 0.001

Model performance of Cognition: R2 = 0.431, adjusted R2 = 0.417, F = 31.911, p < 0.001

Model performance of Courage: R2 = 0.262, adjusted R2 = 0.241, F = 12.675, p < 0.001

Model performance of Patience: R2 = 0.325, adjusted R2 = 0.312, F = 24.178, p < 0.001

## Discussion

This study explores the level of humanistic care ability formental health workers and its potential influencing factors. The authors finds that mental health workers’ humanistic care ability is at a low level. Psychological capital and personality traits are significant predictors of humanistic care ability and its sub-dimensions, but social-demographic variables are not.

Our findings indicate that mental health workers have a low level of humanistic care ability, which is consistent with previous studies [12, 14]. Although people in medical education and clinical practice are increasingly focusing on integrating the concept of humanistic care into personal ability development and clinical work practices, such as the establishment of the patient-doctor relationship, patient treatment and rehabilitation, and colleague relations [2, 27], and also madeprogress. But it is undeniable that medication is still a theme in the field of mental health, ignoring a deeper and interpersonally rich paradigm of understanding and treating mental illness, such as empathetic and humanistic interventions [28, 29]. Furthermore, economic forces and commercial interests now drive the healthcare industry to focus on clinical productivity, efficiency, performance metrics, and regulations, resulting in less time for mental health workers to meaningfully interact with patients and impede humanistic culture [30]. Létourneau et al. [27] also pointed out that the doctors or nurses who have just entered the clinic may voice their desire to provide humanistic care and maintain the ideal of humanistic practice. However, perhaps because of work overload or fear of crossing the ‘professional boundaries’ due to being “too close” to their patient, there is distance between their desires and practice, which hinders the further development of humanistic care ability. It must be mentioned that the “coercion” in psychiatry perpetuates power imbalances in therapeutic relationships, causes mistrust, and exacerbates stigma and discrimination, which may cause patientsto hide their authentic feelings and needs [23]. Mental health workers may become emotionally indifferent due to long-term care of patients with abnormal cognitive function, thus neglecting the patient’s personality, dignity, and satisfaction of needs. As a result, it is difficult for mental health workers to establish a relationship of mutual trust and carry out positive and effective communication with patients. Finally, during the COVID-19 pandemic, mental health workers are faced with more work pressure, workload, or even burnout [31, 32]. They are often powerless and feel difficult to achieve humanistic care. Previous studies [15, 33] have also pointed that we need to reduce work-related stress and burnout to maintain the humanistic spirit and practice.

Interestingly, none of the social-demographic variables in this study predicted the humanistic care ability of mental health workers. Work characteristics including work shift, work pressure, practice environmental satisfaction, salary satisfaction, and work-family conflicts were not statistically significant after entering linear regression. This is different from previous studies. In a previous study [14], there were statistical differences in the humanistic care ability and sub-dimension scores of the medical staff of different ages, education levels, and hospital levels. Although the authors cannot provide evidence-based reasons for this finding, the authors speculate that may be due to the impact of the epidemic, such as changes in the work environment and priorities, service restructuring, remote counseling, measures to control infection risk, anxiety, depression and other negative emotions, and high workload [34], which led to mental health workers having no time to care for their patients during this period. Furthermore, the complexity of the healthcare environment, and sample differences may also contribute to this result. In the follow-up research, this may need to be further explored.

The study notes that psychological capital is positively correlated with humanistic care ability and its two dimensions (cognition and patience). Psychological capital is a positive psychological state during an individuals’ growth and development [35]. It can help individuals adapt to changing demands and demonstrate emotional stability when faced with adversity [35, 36]. Mental health workers with higher levels of psychological capital are more inclined to calmly and confidently solve the obstacles in the humanistic care process, and constantly seek the development of humanistic care ability. Meantime, they are more patient to explore the needs of themselves and others and give care and support to the care recipients. In addition, previous studies have explored the positive effects of psychological capital, such as preventing burnout and reducing the negative effects of work pressure [37]. In other words, psychological capital may also indirectly play a positive role in the development of the humanistic care ability of mental health workers.

Another important finding in this study is that personality traits are significantly associated with humanistic care ability. Among them, the psychoticism is negatively correlated with humanistic care ability and its three dimensions (cognition, courage, and patience); the neuroticism is negatively correlated with humanistic care ability and its one dimension (courage); extroversion is positively correlated with humanistic care ability and its two dimensions (cognition and courage). Personality traits affect the individual’s unique perception and response to the external environment, leading to different results. Extroverts are usually optimistic. They are easy to build harmonious and stable interpersonal relationships at work, and communicate effectively with patients or colleagues [38], to understand the real needs of the care recipients. In addition, extroverts tend to view work positively and are more courageous and responsible when solving various problems in the workplace. They are also more likely to feel more happiness, and this significantly predicts the provision of humanistic care to patients [39]. People with high psychoticism scores may lack sympathy, carelessness, or unkindness to others at work, and cannot integrate well into society or interpersonal relationships. For them, it may be difficult to establish emotional and interpersonal relationships with patients and to listen to the patients’ inner needs, or they may not have the patience to do this. People with high neuroticism scores are emotionally unstable and prone to negative emotional reactions such as anxiety. Their ability to withstand stress is weakened, and tend to amplify the importance of certain situations, thereby experiencing a higher degree of work overload [38], which hinders their humanistic practice to some extent. Also, neurotic medical workers tend to accumulate negative emotions and produce irrational thinking. They may not have the courage or ability to deal with unknown challenges and provide caring behaviors for patients.

This study has several limitations. First, the authors collect the data in a specific area, and whether the results can be generalized to other healthcare systems or territories with a different epidemic situation may need to be further verified by multi-center and large-sample studies in the future. Secondly, the cross-sectional design limits the inferences of causal relationships among the variables, and further longitudinal research may be required. Finally, in self-report questionnaires, the results may be biased.

## Conclusion

The authors find that mental health workers have a low level of humanistic care ability. Managers should focus on the importance of humanistic care for the treatment and rehabilitation of patients with mental illness, and provide support for improving the humanistic care ability of mental health workers and use it effectively in the day-to-day practice of clinical psychiatry. A workflow structure that allows adequate time to establish relationships with patients is critical. The research also finds that psychological capital and personality traits are the significant influencing factors of humanistic care ability and its sub-dimensions. This provides new directions for developing the humanistic care ability of mental health workers, that is, through interventions to improve psychological capital or drive individuals to achieve their desired personality trait changes.

## Data Availability

The datasets generated during and analyzed during the current study are not publicly available due to human subjects’ protections but are available from the corresponding author on reasonable request.

## Abbreviations

CAI: Caring Ability Inventory
PsyCap: Psychological capital
PCQ-24: the Psychological Capital Questionnaire 24
EPQ-RSC: the Eysenck Personality Questionnaire-Revised, Short Scale for Chinese
P: psychoticism
N: neuroticism
E: extroversion
L: lie
